# Patient-Provider Sexual Health Communications and PEP Awareness Among Young Cisgender Men Who Have Sex with Men

**DOI:** 10.1007/s10461-025-05005-1

**Published:** 2026-01-05

**Authors:** Enmanuel Minaya Fernandez, José A. Bauermeister, Katie B. Biello, Lisa B. Hightow-Weidman, Steven P. Meanley

**Affiliations:** 1 School of Nursing, University of Pennsylvania, 418 Curie Blvd, Philadelphia, PA 19104 USA; 2 School of Public Health, Brown University, Providence, RI USA; 3 Institute On Digital Health and Innovation, College of Nursing, Florida State University, Tallahassee, FL USA

**Keywords:** HIV prevention, Sexual health topics, Sexually transmitted infections, Social determinants of health, Health communication

## Abstract

Young men who have sex with men (YMSM) in the United States remain disproportionately affected by HIV, yet their awareness and uptake of post-exposure prophylaxis (PEP) remain suboptimal. Patient-provider communication about sexual health has been shown to influence engagement with HIV prevention services, yet its role in shaping PEP awareness among YMSM not currently using pre-exposure prophylaxis (PrEP) is poorly understood. This study analyzes cross-sectional data from 570 HIV-negative YMSM aged 15–24 to examine associations between provider discussions of sexual health topics and PEP awareness. Multivariable logistic regression models controlled for demographic, behavioral, and structural factors. PEP awareness was associated with prior HIV testing (OR = 2.65, 95% CI: 1.65–4.27), having health insurance (OR = 2.30, 95% CI: 1.20–4.43), prior healthcare access barriers (OR = 1.83, 95% CI: 1.08–3.11), and discussing HIV/STI prevention with a provider (OR = 2.04, 95% CI: 1.32–3.16). Because only HIV/STI prevention discussions, not the broader range of sexual health topics, were associated with PEP awareness, our findings emphasize the value of targeted prevention-focused dialogue between providers and YMSM not on PrEP. Future research should examine how such communication influences not only awareness but also PEP uptake and adherence in this high-priority population.

## Introduction

The ongoing HIV epidemic results in almost 40,000 new HIV infections in the United States (U.S.) each year, particularly among disproportionately affected populations like young men who have sex with men (YMSM) [1–5] Post-Exposure Prophylaxis (PEP) is one of the effective HIV prevention measures used across the world to combat HIV transmission (John et. al 2020; CDC 2016). PEP is a regimen of antiretroviral drugs that begin within 72 h following a possible HIV exposure and continues for a course of 28 days [6]. It was originally used for workplace HIV exposures in the late 1980 s, and later expanded to non-occupational HIV exposures (nPEP) [6].

Despite its effectiveness, PEP awareness, accessibility, and utilization remain suboptimal among disproportionately affected populations. Jin et al., (2022) [7] conducted a systematic review and meta-analysis on the awareness and use of PEP for HIV prevention among men who have sex with men (MSM). The findings indicated that only about 60% of MSM worldwide are aware of PEP, with less than 5% utilizing it. PEP awareness rates vary widely, with higher overall awareness in certain countries including Israel, the U.S., and Europe, but lower in certain areas of China, Portugal, and Spain; notably, despite high general awareness in the U.S., health disparities exist among Black MSM (Jin et al., 2022).The current level of PEP awareness among YMSM in the U.S. who are not using PrEP is underexplored in research efforts. This oversight is critical to address for several reasons. First, PrEP and PEP serve distinct functions in the HIV prevention toolkit. PrEP is a proactive, continuous biomedical intervention whereas PEP is a reactive, time-sensitive strategy used after a potential HIV exposure [8, 9]. Individuals who are actively using PrEP are generally less likely to need PEP. In contrast, those not on PrEP may be most likely to benefit from PEP in the event of a high-risk exposure, making their awareness of this HIV prevention option particularly important for reducing HIV transmission risk. Second, PEP usage can serve as a behavioral indicator suggesting heightened HIV vulnerability. Individuals who have used PEP in the past are often considered ideal candidates for transitioning to PrEP use, assuming they have not seroconverted to HIV, as their PEP use indicates ongoing risk that could be better addressed through continuous prevention [8, 9]. It also represents a critical juncture where health systems can engage and support YMSM with more sustained prevention strategies. Understanding how YMSM not on PrEP perceive and access PEP can thus inform both immediate risk reduction and long-term PrEP uptake efforts.

The specific impact of patient-provider communication on sexual health topics in enhancing PEP awareness and utilization among YMSM not using PrEP has not been thoroughly investigated. Research has demonstrated that effective patient-provider communication about sexual health can improve HIV prevention behaviors among MSM, including YMSM [10]. While existing data highlights the general relationship between provider communication and HIV prevention behaviors in YMSM, there is a notable gap in research specifically examining how such communication influences PEP use among this population [11, 12]. Effective communication between healthcare providers and YMSM patients helps create a comfortable, non-judgmental environment that encourages open and honest discussions about sexual health needs [13]. Furthermore, cultivating effective patient-provider communication where YMSM feel safe discussing their sexual health concerns can increase their access to and utilization of critical HIV preventive services [1].

Addressing these broader social determinants of health and enhancing patient-provider sexual health communication are crucial for improving health outcomes among YMSM at risk for HIV. While the literature underscores the need for multifaceted interventions within HIV/STI prevention to boost PEP awareness and utilization among at-risk populations such as YMSM [1, 13], Jin et al., 2022; [6–9, 14–19] the present study evaluates whether specific sexual health topics, or the cumulative number of topics discussed, are associated with PEP awareness.

This study aims to investigate how patient-provider sexual health communication is associated with PEP awareness among YMSM not currently taking PrEP. Given that PEP access requires a prescription, understanding the characteristics of providers that shape PEP awareness among this group is critical. Insights from this study will offer valuable guidance on how healthcare providers can effectively address health disparities rooted in social determinants and enhance PEP awareness in this population. Consistent with prior literature on the relationship between provider communication and sexual health, we hypothesized that YMSM who engage with healthcare providers on targeted sexual health topics (e.g., sexual dysfunction, sexual pleasure, sexual violence, and sexual orientation) will demonstrate a positive association with PEP awareness. Additionally, in alignment with the social determinants of health literature, we hypothesize that factors such as age, number of sexual partners, racial/ethnic minority status, prior STI diagnosis, healthcare provider access, health insurance status, and participation in discussions about STI and HIV prevention will be associated with higher levels of PEP awareness.

## Methods

The data for this secondary analysis were drawn from the baseline assessment of the Next Generation study, a cross-sectional online study conducted between 2020 and 2021 with N = 800 YMSM between the ages of 15 and 24 in the U.S. The primary aim of Next Generation was to evaluate the acceptability of and challenges using HIV prevention products among YMSM in the U.S. [20].

Details of the study have been previously described [20]. In brief, participants were recruited via targeted advertisements on social media platforms (e.g. Facebook and Instagram) and directed to a study website containing a brief description and a link to an eligibility screener. Inclusion criteria were: (1) assigned male at birth and identifying as male at enrollment, (2) aged 15–24 years, (3) able to read and speak English, (4) reporting same-sex attractions and/or behaviors, and (5) residing in the U.S.

Individuals completed an online screening survey to determine eligibility. We used best practices, including verification of CAPTCHA validations, time to complete the screener, multiple submissions from similar IP addresses, and phone number and e-mail address to identify fraudulent and duplicate responses. Eligible participants were sent an individualized, password protected link where they could complete their electronic informed consent and online survey. We obtained a waiver of parental consent for participation of individuals 15–17 years old. We secured Institutional Review Board approval for all study procedures and materials. Participants received a $40 gift card as compensation for completing the 45-min survey [20].

The original study sample consisted of 800 YMSM; for the purposes of this analysis, we focused on the subset of participants who reported never using PrEP (*n* = 570). Participants excluded from this analysis (i.e., former or current PrEP users) were older (M diff = 0.93, SE = 0.22, *p* < 0.001), had discussed a greater number of sexual health topics with their healthcare providers (M diff = 0.70, SE = 0.09, *p* < 0.001), had a higher prevalence of STI diagnosis (96.9% vs 65.1%, *p* < 0.001), and reported a greater number of sexual partners (M diff = 1.39, SE = 0.33, *p* < 0.001) compared to those not on PrEP. No significant differences were found for race/ethnicity, sexual orientation, health insurance status, or PrEP healthcare barriers between the two groups.

## Measures

### PEP Awareness

PEP Awareness was assessed by asking participants, “Before today, have you heard of people taking anti-HIV medicine after a sexual or drug use exposure, to reduce the risk of getting HIV? This is called post-exposure prophylaxis, or PEP”. Responses were dichotomized as “Yes” (1) or “No” (0).

### Sociodemographic Characteristics

Age was assessed in years (range: 15–24). Race/ethnicity was categorized into four groups: non-Hispanic White, non-Hispanic Black, Hispanic/Latino, or Other Race/Ethnicity. Sexual orientation was measured as a categorical variable and assessed by asking participants their current sexual identity. This variable was recoded into three response options of “Gay,” “Bisexual,” or “Other Sexual Orientation”.

### HIV & STI Related Behaviors

Participants were asked if they had ever had an HIV test? (No, Unsure, Yes). Responses were dichotomized as “No/Unsure” or “Yes”. Prior STI diagnosis was measured by asking if they had ever been diagnosed with an STI by a health professional, with responses being either “No” or “Yes”.

Number of sexual partners in the past 30 days was measured as a continuous variable. Given the skewed distribution of the variable, we recoded it into a categorical variable improve the regression model’s stability and interpretability within our results: no partners, 1 partner, or 2 or more partners.

### Healthcare Access

Health insurance status was assessed by asking participants if they had health insurance or health care coverage, with responses dichotomized as “No Insurance” or “Any Insurance”. Healthcare provider access was assessed by asking participants if there was a time in the past 12 months when they needed to see a doctor or other health care provider but could not. Responses were dichotomized as “Yes” or “No”. Prior healthcare access barriers were measured by asking participants if any of the following factors made it difficult for them to access healthcare: (1) cost, (2) transportation, (3) fear of deportation, (4) fear of mistreatment or harassment by health care providers, staff, or patients, (5) health care providers not understanding their health needs, (6) not knowing how to access a doctor or health care provider, and (7) other (please specify). Responses were “Yes” or “No” for each factor. A composite dichotomous variable was created, indicating “None” if all seven items were “No” and “Any” if any of the seven items were “Yes”.

### Patient-Provider Communication

Patient-provider sexual health communication was measured by asking participants if a healthcare provider had ever talked to them about several sexual health topics: HIV/STI prevention, sexual pleasure, sexual dysfunction, and sexual violence. The number of sexual health topics discussed with a provider was calculated by summing the "Yes" responses across the topics (range: 0–4). Each topic was also examined individually as a dichotomous variable in the regression models. 

## Data Analysis

For this analysis, the sample consisted of 570 YMSM living without HIV and not currently using PrEP. Following a descriptive analysis of our variables, we examined group differences based on participants’ PEP awareness (see Table 1). We then performed two logistic regression models to examine the association of individual and composite patient-provider sexual health communication topics with PEP awareness. The first model (see Table 2) examined the association between the total number of sexual health topics discussed with a provider and PEP awareness, controlling for age, race/ethnicity, prior HIV testing, prior STI diagnosis, prior healthcare barriers and health insurance status. The second model (see Table 2) examined the individual association between each sexual health topic discussed (i.e., HIV/STI prevention, sexual dysfunction, sexual pleasure, sexual violence), while adjusting for the aforementioned covariates. All analyses were conducted using SPSS Version 29.

**Table 1:** Participant characteristics by PEP awareness among young MSM currently not on PrEP (N = 570)

Variable	Full sample (N = 570)	PEP unaware (N = 148)	PEP aware (N = 422)	t-/χ^2^	p
Age, M (SD)	20.9 (2.31)	20.5 (2.51)	21.1 (2.22)	2.70	.007
Race/Ethnicity, *n*(%)					
NH White	245 (43.0%)	62 (41.9%)	183 (43.4%)		
NH Black	82 (14.4%)	26 (17.6%)	56 (13.3%)	2.78	.592
Hispanic/Latino	136 (23.9%)	35 (23.6%)	101 (23.9%)		
Other Race/Ethnicity	107 (18.8%)	25 (16.9%)	82 (19.4%)		
Sexual orientation, *n*(%)					
Gay	403 (70.7%)	104 (70.3%)	299 (70.9%)		
Bisexual	85 (14.9%)	27 (18.2%)	58 (13.7%)	2.66	.264
Other sexual orientation	82 (14.4%)	17 (11.5%)	65 (15.4%)		
HIV testing, *n*(%)					
No/Unsure	149 (26.1%)	65 (43.9%)	84 (19.9%)	32.73	<.001
Yes	421 (73.9%)	83 (56.1%)	338 (80.1%)		
Prior STI diagnosis					
No	470 (82.5%)	129 (87.2%)	341 (80.8%)	3.06	.080
Yes	100 (17.5%)	19 (12.8%)	81 (19.2%)		
Number of sexual partners (past 30 days)					
No Partners	185 (32.5%)	51 (34.5%)	134 (31.8%)		
1 Partner	247 (43.3%0	62 (41.9%)	185 (43.8%)	0.37	.831
2 + Partners	138 (24.2%)	35 (23.6%)	103 (24.4%)		
Health Insurance, *n*(%)					
No Insurance	148 (26.0%)	19 (43.2%)	25 (56.8%)	10.97	.012
Has Insurance	422 (74.0%)	126 (24.3%)	392 (75.7%)		
Prior healthcare barriers, *n*(%)					
None	423 (74.2%)	115 (77.7%)	308 (73.0%)	3.43	.180
Any	124 (21.8%)	25 (16.9%)	99 (23.5%)		
Don’t Know	23 (4.0%)	8 (5.4%)	15 (3.6%)		
Sexual health topics discussed, m(SD)	1.4 (1.1)	1.2 (1.2)	1.5 (1.1)	3.47	<.001
HIV/STI prevention					
No	169 (29.6%)	65 (43.9%)	104 (24.6%)	19.52	.001
Yes	401 (70.4%)	83 (56.1%)	318 (75.4%)		
Sexual dysfunction					
No	520 (91.2%)	139 (93.9%)	381 (90.3%)	1.81	.179
Yes	50 (8.8%)	9 (6.1%)	41 (9.7%)		
Sexual pleasure					
No	527 (92.5%)	134 (90.5%)	393 (93.1%)	1.05	.305
Yes	43 (7.5%)	14 (9.5%)	29 (6.9%)		
Sexual violence					
No	528 (92.6%)	138 (93.2%)	390 (92.4%)	0.11	.741
Yes	42 (7.4%)	10 (6.8%)	32 (7.6%)

**Table 2:** Multivariable logistic regression models examining the association between PEP awareness and provider communication about sexual health (N = 570)

	Model 1				Model 2			
Variable	AOR	95% C.I. Lower	95% C.I. Upper	*p*	AOR	95% C.I. Lower	95% C.I. Upper	*p*
Sociodemographic characteristics								
Age	1.02	0.93	1.12	0.69	1.02	0.92	1.12	0.75
Black race	0.74	0.41	1.36	0.34	0.81	0.44	1.49	0.51
Latino race	1.01	0.60	1.70	0.98	1.07	0.63	1.81	0.82
Other race	1.21	0.69	2.11	0.36	1.26	0.72	2.22	0.42
HIV/STI related behaviors								
HIV testing	2.83	1.77	4.52	0.001	2.65	1.65	4.27	0.001
Prior STI diagnosis	1.18	0.65	2.17	0.87	1.12	0.61	2.06	0.73
Number of partners	1.00	0.85	1.16	0.97	1.00	0.86	1.17	0.98
Healthcare access								
Has health insurance	2.42	1.27	4.63	0.01	2.30	1.20	4.43	0.01
Healthcare provider barriers (Yes)	1.81	1.07	3.06	0.03	1.83	1.08	3.11	0.03
Healthcare provider barriers (Don’t Know)	0.80	0.31	2.05	0.64	0.86	0.33	2.25	0.76
Patient-provider communication								
Cumulative sexual health topics	1.28	0.98	1.67	0.07				
HIV/STI prevention					2.04	1.32	3.16	0.001
Sexual dysfunction					1.77	0.73	4.31	0.21
Sexual pleasure					0.49	0.22	1.07	0.07
Sexual violence					0.90	0.39	2.06	0.79
Constant	0.30			0.24	0.27			0.21

White participants served as the referent category for Race/Ethnicity. Participants without Health Insurance served as the referent group. Participants who did not experience Healthcare Provider Barriers served as the referent category

## Results

### Sample Characteristics

Table 1 presents the descriptive statistics of the overall sample and stratified by PEP awareness. Participants had a mean age of 20.9 (SD = 2.31). Most participants identified as gay (*n* = 403; 70.7%), followed by bisexual (*n* = 85; 14.9%) and other sexual orientations (*n* = 82; 14.4%). Most participants had health insurance (*n* = 422; 74.2%). The sample was racially diverse, with 43.0% (*n* = 245) identifying as non-Hispanic White, 23.9% (*n* = 136) as Hispanic/Latino, 18.8% (*n* = 107) as Other Race/Ethnicity, and 14.4% (*n* = 82) as non-Hispanic Black. A large proportion of the sample (*n* = 470; 82.5%) reported no healthcare access barriers.

Most participants had tested for HIV in the past (*n* = 421; 73.9%) and had not been previously diagnosed with a STI (*n* = 470; 82.5%). Two thirds of the sample reported having one (*n* = 247; 43.3%) or two or more sexual partners in the prior 30 days (*n* = 138; 24.2%).

In terms of patient-provider communication, 70.4% (*n* = 401) reported discussing HIV/STI prevention (Table 1). Fewer participants reported discussing sexual dysfunction (*n* = 50; 8.8%), sexual pleasure (*n* = 43; 7.5%), or sexual violence (*n* = 42; 7.4%) with a healthcare provider. On average, participants discussed 1.4 sexual health topics with a provider (SD = 1.1).

PEP-aware participants were older in age (M = 21.1, SD = 2.22) and discussed more sexual health topics with providers (M = 1.5, SD = 1.1) than those unaware of PEP (Table 1). Furthermore, the majority of participants who were PEP aware had health insurance (*n* = 392; 75.6%), discussed HIV/STI prevention with their provider (*n* = 318; 75.4%), and more likely to have tested for HIV in the past (*n* = 338; 80.1%). We found no bivariate differences in PEP awareness based on race/ethnicity, sexual orientation, history of incarceration, healthcare access barriers, discussion of other specific sexual health topics (sexual dysfunction, sexual pleasure, or sexual violence), number of recent sexual partners, or history of STI diagnosis.

### Correlates of PEP Awareness

The results of the multivariable logistic regression analyses provide insight into the factors associated with PEP awareness among YMSM not currently using PrEP. In Model 1 (see Table 2), cumulative number of sexual health topics did not achieve statistical significance (AOR = 1.28, 95% CI: 0.98–1.67; *p* = 0.07). Prior HIV testing (AOR = 2.83, 95% CI: 1.77–4.52; *p* < 0.001), health insurance (AOR = 2.42, 95% CI: 1.27–4.63; *p* < 0.001), and those reporting healthcare barriers (AOR = 1.81, 95% CI: 1.07–3.06; *p* = 0.03) had a positive association with PEP awareness. No other statistically significant associations were observed with the variables in the model.

In Model 2 (Table 2), HIV/STI prevention health discussions with a provider (AOR = 2.04, 95% CI: 1.32–3.16; *p* < 0.001) was the only sexual heath topic associated with PEP awareness. Prior HIV testing (AOR = 2.65, 95% CI: 1.65–4.27; *p* < 0.001), health insurance (AOR = 2.30, 95% CI: 1.20–4.43; *p* < 0.001), and those reporting healthcare barriers (AOR = 1.83, 95% CI: 1.08–3.11; *p* = 0.03) had a positive association with PEP awareness. No other statistically significant associations were observed with the variables in the model.

## Discussion

Despite the availability and clinical effectiveness of PEP, PEP awareness remains unevenly distributed among YMSM at risk for HIV acquisition. Our findings identify key predictors of PEP awareness and underscore the importance of patient–provider communication on sexual health in promoting PEP awareness among YMSM not using PrEP. Structural conditions (e.g., age, health insurance, health care access barriers), interpersonal dynamics (e.g., discussions with providers regarding sexual health topics), and behavioral factors (e.g., prior HIV testing) are associated with greater PEP awareness among YMSM. This multilevel pattern reinforces the idea that both structural and interpersonal contexts shape how HIV prevention knowledge circulates. These associations carry important implications for programs seeking to integrate PEP into the suite of HIV prevention strategies.

Importantly, our findings point to the role of patient-provider sexual health communication as a modifiable and potentially impactful correlate of PEP awareness. However, the cumulative number of sexual health topics discussed was not associated with PEP awareness in adjusted models. This indicates that topic breadth alone does not explain variation in PEP awareness. Specific discussions about HIV/STI prevention, however, were significantly associated with increased odds of knowing about PEP. Taken together, these findings imply that it is the substance of the communication, rather than its breadth, that is most strongly associated with PEP awareness. Accordingly, our data do not provide empirical support for the idea that covering a wider range of sexual health topics increases PEP awareness. Instead, the association observed in this study is specific to HIV/STI prevention conversations. This is consistent with prior evidence indicating that targeted, risk-specific provider conversations can have more impact on prevention knowledge and behavior than diffuse or generalized counseling [12, 21, 22]. At the same time, HIV/STI prevention discussions among YMSM rarely occur in isolation. Conversations about sexual risk are often interwoven with dialogue about pleasure, sexual functioning, and experiences of sexual coercion or violence,topics that shape how individuals perceive risk, trust providers, and engage with prevention strategies [23, 24]. Future research should examine whether training providers to engage in patient-centered, targeted conversations regarding PEP as part of HIV/STI prevention discussions, as well as situating those discussions within a broader, affirming dialogue about sexual well-being, can increase uptake when clinically appropriate, particularly among youth who are unable or unwilling to use PrEP. Importantly, while such broader dialogues may support trust and engagement, the present findings do not indicate that breadth itself is associated with PEP awareness.

Several social determinants of health (i.e., older age, health insurance status, and prior access to care barriers) were also associated with greater PEP awareness. Given that healthcare access supports engagement with biomedical HIV prevention [25], these findings highlight a concerning disparity: uninsured and structurally marginalized youth may be excluded from essential HIV-related information and services. Notably, participants who reported experiencing barriers to healthcare access in the past were more likely to be aware of PEP. While this finding may seem counterintuitive, it may reflect that youth who encounter barriers are often required to seek prevention information through alternative channels (e.g., peers, online resources, or community organizations). Alternatively, it may capture selective engagement patterns, wherein those previously facing barriers remain more attuned to health system navigation. While this suggests a degree of agency and resourcefulness, it also signals a systemic gap: healthcare systems are not consistently serving as the primary conduit for HIV prevention education, particularly for those disengaged from routine or preventive care [24, 26–28].

Taken together, these findings highlight the need for a more intentional and inclusive approach to HIV/STI prevention among YMSM not using PrEP. Our results suggest that the substance of patient-provider communication, specifically discussions about HIV/STI prevention, rather than the scope of sexual health topics covered, is strongly associated with PEP awareness. In so doing, clinical encounters should emphasize culturally responsive discussions about both PrEP and PEP, acknowledging that individuals may move between prevention strategies over time depending on their life stage, access to care, and evolving perceptions of risk [25]. Providers should foster open and non-judgmental communication with YMSM to create a comfortable environment that encourages discussions about sexual health and HIV/STI prevention [1]. Public health campaigns should work to normalize PEP as a legitimate and accessible HIV prevention option, one that stands alongside PrEP, rather than being viewed solely as a secondary choice. Framing PEP as complementary, rather than competing against other biomedical prevention modalities, may help shift perceptions of its role within combination prevention strategies. Collaborations between healthcare providers, community organizations, and public health agencies should focus on developing targeted outreach, education, and media campaigns to raise awareness about PEP and other HIV prevention options among disproportionately affected populations [15, 29, 30]. Finally, policy efforts should seek to address structural barriers such as insurance gaps, provider stigma, and uneven access to affirming care if we are to close the awareness and utilization gaps that persist across the HIV prevention continuum. This may involve expanding health insurance coverage, improving the affordability and accessibility of PEP, and allocating resources to support community-based HIV prevention initiatives. By implementing interventions at multiple levels, healthcare providers and policymakers can work together to optimize PEP awareness and utilization and help to build trust and continuity between YMSM and healthcare systems, in order to ultimately reduce HIV transmission among YMSM [31, 32].

There are several limitations to this study. First, this study relied on secondary analysis of cross-sectional data, limiting causal inference. Secondly, the self-reported nature of the data introduces the possibility of self-report bias, as participants may have provided responses influenced by social desirability or recall inaccuracies, which could affect the interpretation of the findings. Additionally, the generalizability of this unique sample may not represent all YMSM within the U.S [20]. Additionally, the study did not incorporate other objective measures outside of the variables used in this analysis to assess factors that could influence PEP awareness (e.g., social media engagement, education, housing instability). Furthermore, participants may have interpreted HIV prevention and STI prevention as overlapping topics since HIV is itself an STI, potentially inflating the count of sexual health topics discussed and introducing measurement variability across participants. Given that the observed association between cumulative sexual health topics and PEP awareness was trend-level rather than statistically significant, these findings should be interpreted as suggestive rather than definitive. Replication in larger or longitudinal studies will be important to determine whether this relationship reflects a meaningful pathway or a chance finding. Future research should consider employing longitudinal designs and objective measures to further investigate the impact of patient-provider sexual health communication on PEP awareness.

In conclusion, this study highlights the pivotal role of patient-provider sexual health communication in shaping PEP awareness among YMSM not using PrEP. By ensuring that HIV/STI prevention is routinely and explicitly discussed during clinical encounters, and addressing the structural conditions that shape access to care, PEP can become a more integral component of HIV prevention strategies. While comprehensive sexual health communication remains important for many aspects of care, our findings indicate that targeted HIV/STI prevention dialogue is the element most directly associated with PEP awareness. Increasing its visibility and accessibility, especially among YMSM who are not using PrEP, should be a priority for researchers, practitioners, and policymakers alike in order to close prevention gaps and ultimately reduce HIV transmission among YMSM.
